# A unique case of anti-GBM disease with concomitant anti-PLA2R positivity

**DOI:** 10.1186/s12882-022-02941-1

**Published:** 2022-10-21

**Authors:** Adél Molnár, András Tislér, Deján Dobi, Ákos Pethő

**Affiliations:** 1 Department of Internal Medicine and Oncology, Faculty of Medicine, Semmelweis University, Budapest, Hungary; 2 Institute of Pathology, Forensic and Insurance Medicine, Semmelweis University, Budapest, Hungary

**Keywords:** Membranous nephropathy, Anti-GBM disease, Goodpasture’s syndrome, Glomerulonephritis

## Abstract

**Background:**

Concomitant occurrence of anti-GBM disease and anti-PLA2R positive membranous nephropathy have been previously described. However, to the best of our knowledge, this is the first case report that documents the co-occurrence of the diseases proven by both serologic and histologic methods.

**Case presentation:**

A 51-year-old woman presented to hospital with nausea, bilateral lower extremity edema, dyspnea, dark urine, and then anuria. Symptoms developed one month after an upper respiratory tract infection. Laboratory results showed acute kidney injury, and hypoalbuminemia. Immunologic examination revealed both anti-GBM and anti-PLA2R positivity. Kidney biopsy demonstrated the histological features of Goodpasture’s disease and anti-PLA2R positive membranous nephropathy. Steroid, cyclophosphamide, and plasmapheresis were commenced. Despite the combined immunosuppressive, the patient remained on renal replacement therapy.

**Conclusions:**

Microbial kidney injury can trigger multiple autoimmune diseases. The simultaneous occurrence of anti-glomerular basement (anti-GBM) disease and membranous nephropathy is extremely rare. Delayed recognition leads to delayed treatment, causing worse renal and patient outcomes, as well as increased financial costs.

## Background

Anti-glomerular basement membrane (GBM) disease is an autoimmune disease in which antibodies are formed against the type IV collagen and presents with rapidly progressive glomerulonephritis [1]. Membranous nephropathy (MN) is also an autoimmune-mediated glomerular disorder wherein podocyte and capillary wall injury result in proteinuria and nephrotic syndrome. Primary MN is associated with antibodies against phospholipase A2 receptor (PLA2R) in about 80% of cases [1].

Simultaneous occurrence of anti-GBM disease and membranous nephropathy is rare but documented [2–8]. However, to the best of our knowledge, anti-glomerular basement disease and concomitant anti-PLA2R positive membranous nephropathy confirmed by serology and histology has not been published yet. We report a case of a patient with concurrent clinical, serologic, and histological features of both disorders.

## Case presentation

A 51-year-old Caucasian woman with an unremarkable medical history developed cloudy and subsequently brown-colored urine on 25th September 2021. The patient received ciprofloxacin treatment for a suspected urinary tract infection.

**Fig. 1:**
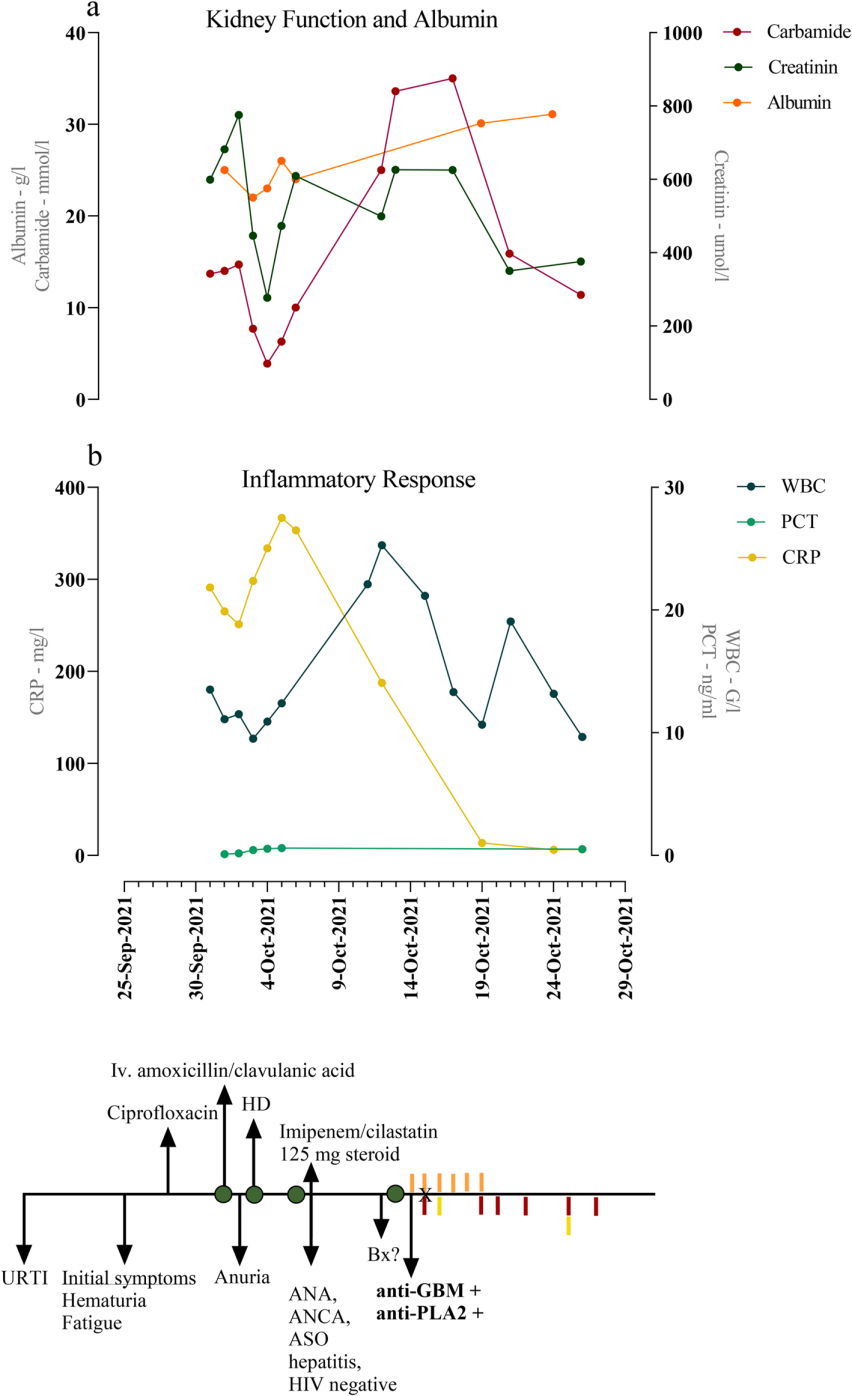
Summary of the main laboratory parameters, symptoms, diagnostic, and therapeutic steps. **a** Kidney function and albumin level over time. **b** Inflammatory parameters over time. Significant symptoms, diagnostic and therapeutic interventions are summarized in the timeline. Green circles: hospital admissions, orange columns: iv. administration, red columns: plasmapheresis, yellow columns: cyclophosphamide administration, X: the day of the biopsy. *Iv* intravenous, *URTI* upper respiratory tract infection, *ANA* anti-neutrophil antibody, *ANCA* anti-neutrophil cytoplasmic antibody, *ASO* anti-streptolysin O, *HIV*: human immunodeficiency virus, *Bx* biopsy, *anti-GBM* anti-glomerular basement membrane, *anti-PLA2R* anti-phospholipase A2 receptor, *HD* initiation of hemodialysis, *WBC* white blood cell, *PCT* procalcitonin, *CRP* C-reactive protein [Image was created with GraphPad (GraphPad Prism 9.0.0)]

Subsequently, she developed oliguria, edema, resting dyspnea, vomiting, and diarrhea. In light of these emerging symptoms, she was admitted to a secondary care hospital. Initial laboratory evaluation showed anemia, high C-reactive protein (CRP) without procalcitonin (PCT) elevation, and mild leukocytosis (Fig. 1).

Urine dipstick testing found hematuria and proteinuria; automated sediment revealed leukocytes and erythrocytes but no bacteria. Arterial blood gas analysis showed mixed respiratory and metabolic acidosis. Blood and urine cultures were negative. Ultrasonographic findings showed bilateral renal enlargement and increased parenchymal echogenicity.

The suspected diagnosis was acute kidney injury due to possible septicemia; hence initial treatment with empirical intravenous amoxicillin/clavulanic acid was initiated. Nevertheless, soon she became anuric, and laboratory abnormalities also worsened, hence hemodialysis was commenced. At this point, the patient was transferred to a nephrology unit department for further evaluation.

Post-infectious glomerulonephritis, vasculitis, renal arterial or venous thrombosis was suspected as differential diagnoses. Due to the high CRP, empirical imipenem/cilastatin, and intravenous methylprednisolone (125 mg), were initiated. Anti-nuclear antibody screening test, anti-neutrophil cytoplasmic antibodies, and anti-streptolysin O serology were negative. Complement levels were normal. Hepatitis A, B, C, E, and HIV infections were ruled out. Thrombosis could not be proved either. Chest X-ray demonstrated pleural effusion and basilar pulmonary edema, which also was confirmed by non-enhanced chest CT.

In order to perform a renal biopsy, the patient was transferred to our nephrology division at the Department of Internal Medicine and Oncology.

Upon admission to our department, we could not reveal any nephrotoxic medication or substance use. Zoonotic infection was not probable. She lived a physically active lifestyle but did not exert herself beyond normal. Nonetheless, two weeks before her urinary discoloration, she had had an upper respiratory tract infection.

Laboratory findings on admission showed anemia, leukocytosis, and secondary hyperparathyreosis. Immunologic testing revealed circulating anti-GBM antibody level of 2822 CU, and anti-PLA2R titer of 1:320 (Fig. 1).

**Fig. 2:**
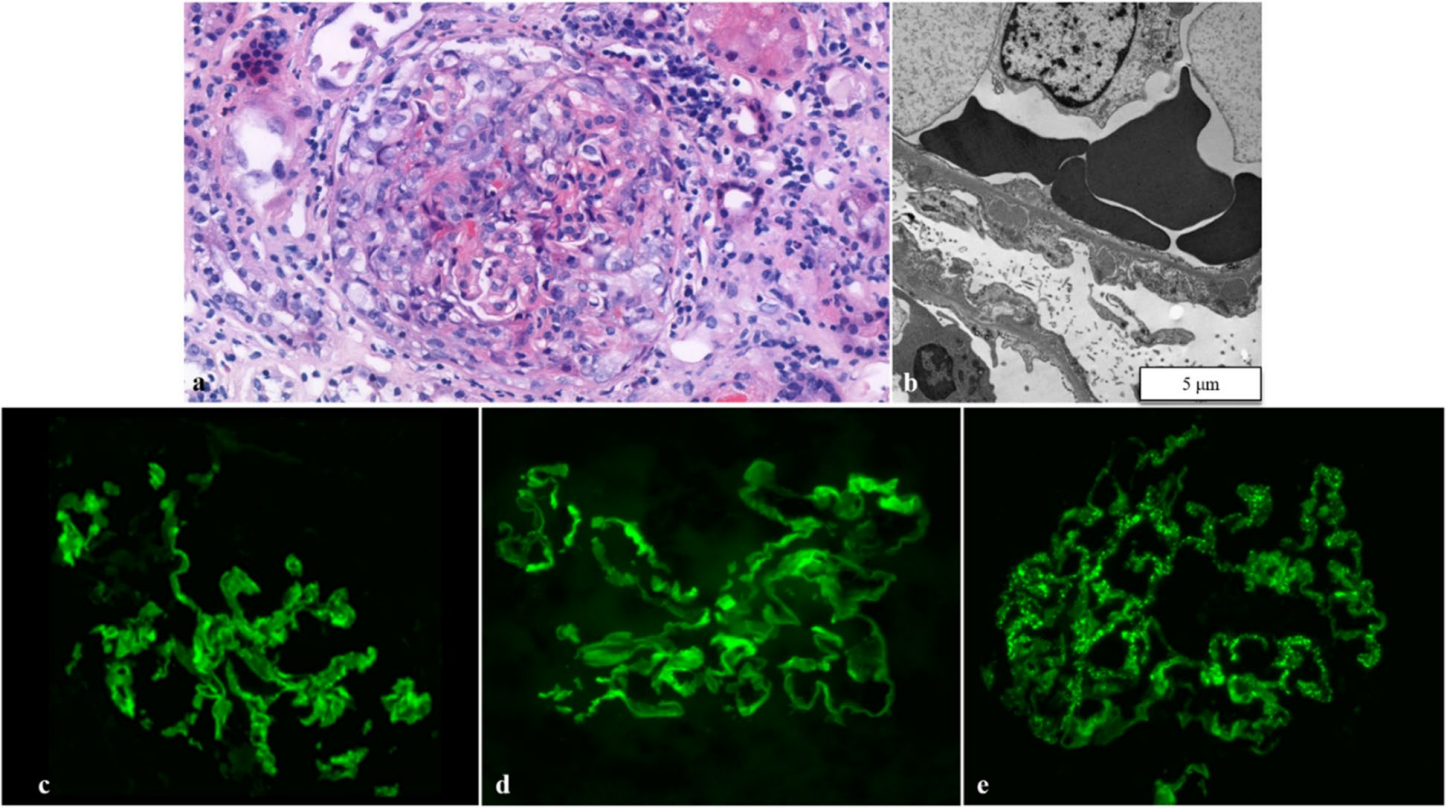
**a** Cellular crescent, hematoxylin and eosin stain, 200x. **b** Transmission electron microscopic image, with diffuse foot process effacement of the podocytes and subepithelial immunocomplex deposits 8000x. **c** Direct immunofluorescence reaction with FITC (fluorescein isothiocyanate) labeled anti-IgG (note the simultaneous granular and linear staining). **d**, Direct immunofluorescence reaction with FITC labeled anti-IgG3 (linear staining). **e** Direct immunofluorescence reaction with FITC labeled anti-PLA2R (granular staining)

This rare serologic constellation prompted a renal biopsy. The total number of obtained glomeruli was 16, of which one was globally sclerosed, and 14 showed cellular crescent formation some with fibrinoid necrosis and capillary wall rupture. In some other glomeruli, Bowman’s capsule integrity was also disrupted. Advanced interstitial fibrosis/tubular atrophy could also be observed. Immunofluorescence microscopy demonstrated both linear and granular depositions of immunoglobulin G, kappa and lambda light chains, and C3. Staining with PLA2R appeared as granular signal. One non-crescentic glomerulus was available for electron microscopy that showed Ehrenreich-Churg III-IV stage subepithelial immunocomplex deposits (Fig. 2).

Based on the clinical and histological findings, the patient was diagnosed with anti-GBM glomerulonephritis and concomitant PLA2-R positive membranous nephropathy.

Based on these findings we initiated prompt plasmapheresis and induction therapy with intravenous methylprednisolone. From day three, she continued to receive pulse cyclophosphamide fortnightly.

During her inpatient stay, she underwent six plasmapheresis sessions and received in total of 4.5 g intravenous methylprednisolone, and 1250 mg cyclophosphamide. Steroid was continued and tapered orally. Subsequently, anti-GBM antibody titer decreased to 424 CU, and anti-PLA2R became negative. Despite all this aggressive treatment, her renal function did not improve, and she remained dialysis-dependent. At present living-donor kidney transplantation is planned after her immunological remission.

## Discussion and conclusion

In this report, we presented a patient with rapidly progressive glomerulonephritis, anti-GBM disease that was established by demonstrating the anti-glomerular basement membrane antibody in the sera, and linear immunofluorescence staining on the biopsied specimen. The serum anti-PLA2R antibodies and granular staining with the antibodies upon immunohistochemistry revealed membranous nephropathy as well.

A small number of patients with anti-GBM disease present with nephrotic syndrome, and in extremely rare cases, crescents can also be seen in primary membranous nephropathy [6, 7]. The simultaneous occurrence of the two entities has also been published in a small number of cases. However, this is the first report on serum and histologic anti-PLA2R positivity.

Previous studies proposed a sequential development of the two diseases. Experimental animal studies hypothesized that immune complex deposition can unmask concealed antigens or alter endogenous epitopes [5, 6], but the initiating target antigen and the exact process still need to be elucidated. There is no consensus whether anti-GBM or anti-PLA2R antibody deposition happens first either [3].

The absence of anti-PLA2R positivity in the concomitant disease might be due to the lack of testing. Only two studies have mentioned PLA2R positivity on anti-GBM biopsy specimens [2, 9]. In these cases, immunohistochemistry was conducted after discovering the histologic characteristics of membranous nephropathy, but serum investigation had not been performed before, nor after the biopsy. This is likely due to the fact that the symptoms suggest predominantly anti-GBM disease that does not necessitate the test for anti-PLA2R. The discovery of the concomitant disease was accidental in all cases. It has also been suggested that anti-PLA2R antibodies might be cleared from the serum quickly due to glomerular deposition, so they cannot be detected in the serum [10]. On the other hand, the lack of serum positivity in literature can also be attributed to the relatively new utilization of anti-PLA2R antibody [11].

In our patient, the presence of nephrotic range proteinuria before the anuric phase led us to investigate the anti-PLA2R titer. The rapidly progressive course of the disease suggested screening for the anti-GBM antibodies. Although these antibodies are pathognomic for both entities, none of them is 100% specific and sensitive; both false-positive and false-negative cases can occur [1, 12, 13]. This, and the odd findings, impelled us to perform the kidney biopsy to confirm the diagnoses. It also aided our work to predict kidney- and patient outcomes.

There are no precise recommendations on the therapy for this scenario. Upon reviewing the previous reports, we launched the treatment according to the KDIGO recommendations on anti-GBM disease [14].

Besides immunosuppressive therapy and plasmapheresis, the prognosis is primarily dependent on rapid diagnosis. Sclerosis and its extent are associated with chronicity: the longer the time from the diagnosis, the worse the prognosis is. Hyalin thrombi on biopsy correlate with progression to end-stage renal disease [15]. Literature review shows a poor prognosis in patients with dual glomerulopathy, which is also supported by our experience [2, 16].

Although serologic tests are widely available, delayed diagnosis and transferring of patients to tertiary centers are common in the management of anti-GBM vasculitis [17]. This partly relates to the rarity of the entity, thus the decreased awareness. Diagnosis can also be challenging due to the misleading clinical features which may be explained by an alternative diagnosis, thus deferring serologic testing. Rarely, delayed diagnosis may be attributable to the lack of sensitive bioassay use, resulting in false negativity [12]. Delayed recognition leads to delayed treatment, causing worse renal and patient outcomes, as well as increased financial costs [18].

We described a case of concurrent anti-GBM disease and anti-PLA2R positive membranous nephropathy. The exact mechanism of the transition from one to the other remains unknown. Anti-GBM vasculitis superimposed with another glomerular disease results in faster renal function deterioration, leading to end-stage renal disease. Thus, early recognition and prompt treatment would be the only possibility to preserve kidney function. Our findings and the previous case reports of the concomitant disease suggest that this subset of patients have different prognostic implications, and a more aggressive therapy might be needed.

### Availability of data and materials

Data sharing is not applicable to this article as no datasets were generated or analysed during the current study.

## Data Availability

Aggregate patient data from the authors upon reasonable request; patients are unidentifiable.

## Abbreviations

CRP: C-reactive protein
CT: Computed tomography
GBM: Glomerular basement membrane
KDIGO: Kidney Disease Improving Global Outcomes
MN: Membranous nephropathy
PCT: Procalcitonin
PLA2R: Phospholipase A2 receptor
